# Compassionate care from the perspective of the hospitalized patient

**DOI:** 10.15649/cuidarte.5399

**Published:** 2026-07-09

**Authors:** Francy Edith López Herrera, Mayerly Andrea González Duque, Mariana Zapata Pineros, María Camila Guzmán Vargas, María Clara Vélez Ángel, Alicia Krikorian

**Affiliations:** 1 Care Group, School of Health Sciences, Universidad Pontificia Bolivariana, Medellín, Colombia. E-mail: francy.lopez@upb.edu.co Universidad Pontificia Bolivariana Madellín Colombia francy.lopez@upb.edu.co; 2 School of Nursing. Universidad Pontificia Bolivariana, Medellín, Colombia. E-mail: mayerlygonzalezd@gmail.com Universidad Pontificia Bolivariana Madellín Colombia mayerlygonzalezd@gmail.com; 3 School of Nursing. Universidad Pontificia Bolivariana, Medellín, Colombia. E-mail: mzapatap10@gmail.com Universidad Pontificia Bolivariana Madellín Colombia mzapatap10@gmail.com; 4 Clínica Universitaria Bolivariana. Universidad Pontificia Bolivariana, Medellín, Colombia. E-mail: mariac.guzman@upb.edu.co Universidad Pontificia Bolivariana Madellín Colombia mariac.guzman@upb.edu.co; 5 Pain and Palliative Care Group, School of Health Sciences, Universidad Pontificia Bolivariana, Medellín, Colombia. E-mail: maclavelez@gmail.com Universidad Pontificia Bolivariana Madellín Colombia maclavelez@gmail.com; 6 Pain and Palliative Care Group, School of Health Sciences, Universidad Pontificia Bolivariana, Medellín, Colombia. E-mail: aliciakriko@gmail.com Universidad Pontificia Bolivariana Madellín Colombia aliciakriko@gmail.com

**Keywords:** Health Personnel, Inpatients, Professional-Patient Relations, Empathy, Personal de Salud, Pacientes Internos, Relaciones Profesional-Paciente, Empatía, Pessoal de Saúde, Pacientes Internados, Relações Profissional-Paciente, Empatia

## Abstract

**Introduction:**

Compassionate care is essential for the quality of care. However, few studies in our context address patients' perspectives on compassionate care.

**Objective:**

To explore patients' perceptions of the elements that constitute compassionate care.

**Materials and Methods:**

A qualitative approach using interpretive phenomenology. The participants were hospitalized adults. Convenience sampling was used, with the sample determined by data saturation. Semi-structured interviews were recorded, with prior informed consent and signed consent. Analysis was carried out simultaneously with data collection, through the researchers' interpretation. Units of meaning were identified for each participant and subsequently grouped according to common meanings.

**Results:**

Thirteen hospitalized patients participated, interviewed between January and June 2023. The themes that encompass the meaning of compassionate care were Healing Presence, Compassion: An Experience of Human Connection, Compassion as a Holistic Phenomenon of Caring, and The Antonyms of Compassionate Care.

**Discussion:**

The topics are related to research findings from different contexts and clinical care settings. Differences and similarities are highlighted, broadening the scope of the phenomenological interpretation of each case.

**Conclusion:**

Patient education and communication are central elements of compassionate care, enabling meaningful and humanized relationships in care. The way healthcare providers communicate information and interact with patients directly influences their perception of the care receive.

## Resumen

**Introducción:**

El cuidado compasivo es esencial para la calidad asistencial. Sin embargo, pocos estudios en nuestro contexto abordan la perspectiva de los pacientes sobre el cuidado compasivo.

**Objetivo:**

Explorar la percepción que tienen los pacientes sobre los elementos que configuran un cuidado compasivo.

**Materiales y métodos:**

Enfoque cualitativo desarrollado como fenomenología interpretativa. Participaron adultos hospitalizados. Se empleó un muestreo por conveniencia, con un número determinado por saturación de los datos. Se realizaron entrevistas semiestructuradas grabadas, previo consentimiento informado y firmado. Análisis desarrollado simultáneamente a la recolección de información, a través de la interpretación de los investigadores. Se identificaron unidades de significado de cada participante que posteriormente se agruparon según significados comunes.

**Resultados:**

Participaron 13 pacientes hospitalizados, entrevistados entre enero y junio de 2023. Los temas que agrupan el significado del cuidado compasivo fueron: Presencia que sana, la compasión: una experiencia de conexión humana, La compasión como fenómeno holístico del cuidado y los antónimos del cuidado compasivo.

**Discusión:**

Los temas se relacionan con hallazgos investigativos de diferentes contextos y entornos de atención clínica. Se destacan diferencias y similitudes que amplían el espectro de la interpretación fenomenológica realizada de cada caso.

**Conclusiones:**

La educación y la comunicación para los pacientes son elementos centrales del cuidado compasivo, permiten establecer vínculos significativos y humanizados en la atención. La forma en que el personal de salud transmite información y se relaciona con los pacientes influye directamente en la percepción del cuidado recibido.

## Resumo

**Introdução:**

O cuidado compassivo é essencial para a qualidade da assistência à saúde. No entanto, poucos estudos em nosso contexto abordam as perspectivas dos pacientes sobre o cuidado compassivo.

**Objetivo:**

Explorar as percepções dos pacientes sobre os elementos que constituem o cuidado compassivo.

**Materiais e métodos:**

Foi desenvolvida uma abordagem qualitativa como fenomenologia interpretativa. Os participantes foram adultos hospitalizados. Utilizou-se amostragem por conveniência, com o número de participantes determinado pela saturação dos dados. Entrevistas semiestruturadas foram gravadas após a obtenção do consentimento livre e esclarecido por escrito. A análise foi conduzida concomitantemente à coleta de dados, por meio da interpretação dos pesquisadores. Unidades de significado foram identificadas para cada participante e, posteriormente, agrupadas de acordo com significados comuns.

**Resultados:**

Treze pacientes hospitalizados participaram, entrevistados entre janeiro e junho de 2023. Os temas que agruparam o significado de cuidado compassivo foram: Presença Curativa, Compaixão: Uma Experiência de Conexão Humana, Compaixão como um Fenômeno Holístico do Cuidado e Antônimos de Cuidado Compassivo.

**Discussão:**

Os temas abordam resultados de pesquisas em diferentes contextos e ambientes clínicos. Diferenças e semelhanças são destacadas, ampliando o escopo da interpretação fenomenológica de cada caso.

**Conclusões:**

A educação e a comunicação com os pacientes são elementos centrais do cuidado compassivo, possibilitando o estabelecimento de relações significativas e humanizadas no cuidado. A forma como a equipe de saúde transmite informações e interage com os pacientes influencia diretamente a percepção do cuidado recebido.

## Introduction

Health problems that lead to hospitalization often have a significant impact on individuals, generating physiological, psychological, and even social and spiritual changes. This occurs because of the illness itself and how it is perceived, the conditions of hospitalization, lack of knowledge, uncertainty, separation from the usual environment, and treatments^1^. Added to this are the characteristics of the relationships established with healthcare staff, where depersonalization of care frequently occurs, as it tends to focus more on the disease than on the person suffering from it^1^,^2^. From the perspective of the person-centered care model, it is proposed that care should focus on aspects that go beyond illness and be understood as a driving force for humanization that should permeate healthcare. Thus, compassionate care, as part of humanized care, becomes central to hospital care^3^.

The hospital setting is a complex environment where experiences of well-being and suffering converge, affecting the whole person^1^. The relationships between patients and healthcare staff give rise to diverse experiences, perceptions, and emotions, which significantly influence the quality of care provided^2^. When dehumanized and depersonalized care focused on the illness occurs, communication and empathy in the therapeutic relationship are impaired, which can negatively impact the patient's experience and recovery process^3^. Conversely, compassion in care has been shown to be associated with greater patient satisfaction, quality of life, and recovery, as well as better therapeutic relationships^2^,^4^.

Compassionate care in the hospital setting not only involves concrete actions related to the diagnosis and treatment of people's morbid conditions, but also includes an empathetic attitude of understanding and full attention towards the patient and their family; in this regard, compassion is defined as “a virtuous response that seeks to address the suffering and needs of the person through understanding and relational actions”^4^. Compassion stems from the recognition of the patient as a unique individual with diverse needs, emotions and experiences that, when identified and managed through the care process, contribute to comprehensive care and increased satisfaction with the care received^5^.

Compassionate care not only benefits the patient and their family, but also the healthcare staff, by reducing their levels of stress and emotional exhaustion associated with clinical care^6^. However, it has been shown that healthcare professionals face challenges related to compassion fatigue and emotional burnout, which can hinder the provision of compassionate care and, therefore, negatively affect the hospital experience of patients^7^,^8^.

From a healthcare perspective, compassionate care is considered an intrinsic part of ethics for healthcare professionals and is regarded as a hallmark of quality care and a patient’s right^6^,^9^; unfortunately, in many cases, the compassionate care patients receive is insufficient, affecting their well-being, satisfaction, and perceived quality of care^9^. Hence, the importance of healthcare systems in each context and culture, and the various entities that comprise them, understanding what patients mean by compassionate care, with a view to establishing strategies to promote it and establish it as part of the institutional and relational culture.

Evidence regarding the perceptions of care held by different actors in the healthcare process, as well as the impact of compassionate care, comes primarily from Anglo-Saxon or developed countries, according to a recent systematic literature review^9^. Few studies exist from Spanish-speaking contexts, and even fewer from Latin American countries. Many of these studies focus on aspects such as empathy rather than specifically compassion, or include non-clinical populations (e.g., students)^9^. In Colombia, the available literature is mainly focused on community experiences^10^,^11^ and perceptions from nursing staff^12^. To our knowledge, no studies have been conducted in our context considering our cultural characteristics to understand what those who receive compassionate care understand by it, which differs from findings in studies from other regions. Therefore, this study seeks to explore patients' perceptions of the elements that constitute compassionate care in a tertiary care institution in Medellín, Colombia, based on their experiences interacting with healthcare staff during their hospitalization. The aim is to propose actions to promote compassionate care in the hospital setting.

## Materials and Methods

**Type of study**

This qualitative research study was developed as an interpretive phenomenology that integrates three philosophical traditions: phenomenology, hermeneutics, and ideography to develop a systematic method that analyzes how people give meaning to significant experiences, through a double hermeneutics and a deep approach to each case^13^. Through this method, the study sought to interpret the perception of compassionate care held by hospitalized individuals in a high-complexity health institution in the city of Medellín (Colombia), which has an internal humanization policy and is a reference point for all municipalities in the department of Antioquia.

**Participants**

The study population consisted of 13 individuals hospitalized in a tertiary care institution during the first half of 2023. Inclusion criteria were being hospitalized for at least three days and being an adult, willing to participate voluntarily. Exclusion criteria included: individuals with neurological impairment that limited their participation, those under isolation, those with a serious illness, those with difficulty communicating verbally, or those who did not understand Spanish.

**Data collection instrument and procedure**

Participant recruitment was conducted using convenience and purposive sampling. Lead nursing professionals in inpatient services were contacted by patients who met the eligibility criteria. After receiving approval from the professional, the researchers met with the patient and their companion in the patient's room, explained the study, its objectives, purpose, and potential benefits for continuous improvement of care. The patient was then invited to participate, and if they agreed, the informed consent form was provided. After reading it and addressing any questions, the patient and their companion were asked to sign the consent form. The patient's privacy, comfort, and health status were considered. It was clarified that they could end the interview at any time if they wished and that the information obtained would be safeguarded by the researchers and the names would not be disclosed. All interviews were conducted by MCG (MSc), a practicing psychologist with clinical and research training. The initial interviews were assisted by psychosocial professionals (MCV and AK) and a nurse (FL), all with experience in research interviews.

The semi-structured interviews were conducted using a guide of open-ended questions designed to initiate the conversation: (1) What does compassionate care mean to you? (2) Do you believe that the care you have received during this hospitalization has been compassionate? (3) What do you do or what would you do to make the care you receive compassionate? (4) During your current hospitalization, what experiences have you had that you would describe as compassionate care? (5) During your current hospitalization, what experiences have you had that do not constitute compassionate care? (6) What do you believe are the effects on your well-being of receiving, or conversely, not receiving compassionate care during your hospitalization? With some participants, further questions arose to deepen the understanding of emerging themes. The interviews lasted between 30 and 45 minutes, were recorded, and then transcribed. Aspects such as nonverbal language, pauses, silences, changes in tone of voice, and emotional responses were recorded, as well as the times they occurred. An identification code was established for each interview to preserve the participant's anonymity.

**Analysis**

The analysis was developed concurrently with data collection. The researchers interpreted each participant's significant experience, based on Interpretive Phenomenological Analysis (IPA)^13^.

Each interview was transcribed into a separate Word file, named with the code assigned to each participant. The transcription was done by MAG and MZ, who did not participate in the interviews. Subsequently, each researcher carefully reviewed each interview, identifying key ideas and concepts that allowed them to interpret each participant's significant experience of compassionate care. The researchers then shared their findings with each participant, which helped identify similar interpretations and reduce related biases.

Based on the themes that emerged from the researchers' interpretation and the similarities in the findings and testimonies, each participant's results were grouped into overarching themes that best represented their perception of compassionate care. Subsequently, according to the similarities and commonalities among the themes and meanings found among the participants, the researchers defined four overarching themes that represented the perception of compassionate care for all the patients who participated in the study. Finally, they described each participant's perception of compassionate care within the framework of these four overarching themes resulting from their interpretation.

It was not possible to share the transcripts with the participants to adjust, given their discharge from the healthcare facility and the fact that most of them resided in other municipalities. The interview did not include the patient's contact information. However, during the interview, the patient's responses were paraphrased to confirm the interviewer's initial interpretation.

The study's rigor criteria were applied across all stages to strengthen the internal and external validity of the results: (1) credibility, achieved by establishing trusting relationships with participants and triangulating researchers throughout the research process; (2) transferability, describing the conditions under which the research was conducted to allow other researchers to assess the applicability of the findings in similar contexts; (3) reliability, documenting the research process in analysis matrices that allow for external auditing and replicability of the study; (4) confirmability, using triangulation of disciplinary perspectives from psychology and nursing to enrich the understanding of the phenomenon from different approaches to care^14^.

The study was approved by the Health Ethics Committee of the Universidad Pontificia Bolivariana (minutes 3 of 2022), and participants signed informed consent forms. According to Resolution 8430 of 1993 (Colombia), it was classified as minimal risk^15^. Bioethical principles were respected and applied throughout the research process. All collected data are freely available for access and consultation on Mendeley Data^16^.

## Results

All participants invited to the study agreed to participate (n=13). On average, they were 45.77 years old and had been hospitalized for an average of 13.62 days. Their demographic, social, and health characteristics are described in Table 1.

**Table 1:** Demographic, social and health characteristics of the participants

ID	Sex	Age	Socioeconomic level	Days of hospitalization	Reason for hospitalization
1	Female	47	Medium low	8	Genitourinary disorders
2	Female	56	Half	22	Pulmonary alterations
3	Male	38	Low	7	Gastrointestinal disorders
4	Female	39	Medium low	5	Gastrointestinal disorders
5	Female	59	Medium low	3	Gastrointestinal surgeries
6	Female	53	Medium low	12	Cardiovascular disorders
7	Male	55	Low	15	Pulmonary alterations
8	Male	60	Half	23	Pulmonary alterations
9	Female	20	Half	22	Gastrointestinal disorders
10	Female	45	Low	5	Gastrointestinal disorders
11	Female	37	Low	8	Genitourinary disorders
12	Female	36	Medium low	17	Pelvic surgery
13	Female	50	Low	30	Metabolic disorders

The analysis yielded four themes or constituent elements of compassionate care: “Healing Presence”, “Experience of Human Connection”, “Holistic Phenomenon of Care”, and “Antonyms of Care”, resulting from the researchers' interpretation of each participant's individual perception of compassionate care.

The feature that became one of the central themes, emerging from the personal experience of several patients, and encompassing a diversity of concepts and constitutive aspects of compassion, was: “Healing Presence”.

For one of the patients, feeling that the healthcare staff is attentive to their progress, their needs, and the result of their treatment translates into feelings that make their hospital stay more positive. While an immediate recovery doesn't occur, these attitudes do help them feel supported and cared for. As they explain:

*“They care a lot about the patient, more than they should…very good care, very attentive. “How are you feeling? How are you or how do you feel about the baby?” Everyone is very attentive…the way and the effort they put into caring for the patient, making the patient feel accompanied, making the patient feel well, I think” (FF5, DD6)*

The healing presence means that healthcare staff demonstrate interest in and attentiveness to the patient's recovery process, their symptoms, and any potential risks associated with their treatment. This might be considered a normal process, part of what should be or should be done, but when healthcare staff express this interest in connecting with the other person and care about how they feel, this interest is perceived by the patient as a genuine presence that facilitates their journey toward recovery. This is how they express it:

*“I mean, they’re very attentive. … They always ask me if I’m in pain, how I feel, how I’m doing. If I’m going to surgery, they wish me well, they’re very attentive when I get back from surgery… They worry that I’m okay or that I’m not going to have a drug relapse and all that, well, they come and check on me every day to make sure I’m okay and… you know what I mean?... (GG 4,6,2.)*

Compassionate care is understood by another participant as the presence of staff, which is not limited to the number of activities typical of a hospital setting. For him, this presence manifests as a constant concern for the patient's well-being and the prevention of any risk that could harm him; in this way, the entire care team exerts a healing presence. This is how it is interpreted in the following testimony:

*I mean, even though they're busy with their own things, they don't forget that there's a sick person here. I think that's really nice... And they're always thinking, "Look, be careful not to drop it, it's wet here," "Okay, be careful, look at this," "Remember that..." You know what I mean? For me, all of that is done with care, and not just by the head nurse, but even the cleaning lady, even the orderly, they're concerned about the smallest things. They come and say, "Are you going to stay with her?" "Well, I hope you're well soon," you know, "Have a good night." (KK 9,20,24)*

Another theme that emerged from the analysis of some patients' perceptions of compassionate care is " Compassion, an experience of human connection." For them, the presence, and especially the way in which the healthcare staff interacts with them during their care and recovery process, goes beyond simply fulfilling their job duties.

One of the participants senses that the behavior of the healthcare staff not only responds to the tasks inherent in their work but goes beyond that; he perceives it as a genuine feeling of connection with their needs that increases his satisfaction with the care, because it makes him feel valued. He expresses it this way:

*“Compassion is like concrete love at work. The love with which they arrive every morning or every night. “How are you? Are you in pain? I’m going to give you this medicine, we’re also going to take these pills,” it’s very close care, yes, they have their profession, but in addition to that, they show affection to one… (HH 9,7,6)*

Compassion is understood as an experience of human connection that always manifests itself, especially when the patient is most vulnerable, making them feel like a person regardless of the care that must be provided to achieve this. This is how one of the participants perceives it:

*…Another thing that makes you say “wow,” when they’re going to clean you up and it’s like, with that love or respect, like, not like, just like that, never, it’s always “Hey, relax, I’m here for that, I’m going to help you a lot, that’s doing things with love. (DD 7,6)*

Compassion, in turn, is understood as an experience of human connection insofar as it involves individualized care that considers personal history. This care is not only defined for each case but is also adapted to the individual's particularities, prior experiences, needs, fears, and expectations. This, in turn, is not possible without a genuine interest in the well-being of the other person. This is how the experience recounted by one participant is interpreted:

*“I feel cared for because there’s something very beautiful and special here, and that is that for me, this hasn’t been an easy pregnancy after the death of my son, with the fear that the same thing would happen to me. So, what’s beautiful here is that they understand, they care a lot about making sure you feel good, that the baby is okay. I think it’s a very beautiful hospital in that sense…giving you strength, making you feel heard, understood…because the strength they’ve given me is immense. The way they treat me, allowing Juan to be by my side here, makes me feel very good. I’m truly happy here, I really love it for that reason (EA 6,4,18,35)*

Compassion, understood as an experience of human connection, was interpreted by one of the patients as closeness and familiarity with the healthcare staff, especially when he perceived gestures that evoked his relationship with his own family. This is how one of the participants described it:

*“When they hear the baby, they are very loving, they say: ‘Where are you going?’ Then they find him and they always do it in the best way, they never look unhappy, no it is as if they were not doing it for someone they know, it is as if they were doing it for a child, for a sister, a family member, they do it with love and passion, that is the most important thing. (EF 4,9,10)*

Another theme that describes the patient's perception is compassion as a holistic phenomenon of care. Holistic in that it transcends the satisfaction of basic care needs, which is generally and homogeneously addressed; it focuses on the human aspect, on a genuine interest in contributing to other realities that afflict the patient and hinder their recovery. One of the participants perceives it this way:

*“For my health, it has really helped me a lot emotionally as well, because when you're emotionally well, even your body feels better. When you feel calm and happy, everything seems to flow. Yes, because sometimes you're very sad, and your platelet count drops, your spirits plummet, and everyone has been interested in helping me with that, too. (EE6, AA4)*

For another participant, compassion is a holistic aspect of care, insofar as it considers the patient's family as part of the attention they receive. Showing interest in the family's well-being is significant for the patient because it minimizes the worries that arise from hospitalization. As they explain:

*“The comfort of the companion, yes, the one who comes to take care of you, that they make sure he can accompany me, that they give him a chair so he is more comfortable, that was very good… because he had nowhere to stay and that made me feel bad…” (CC 4 DD 2,23)*

The antonyms of compassionate care represent another interpretation of the term for patients, understood as impersonal or dehumanized care provided without regard for emotional, spiritual, or personal needs. One patient's experience suggests that not being listened to by healthcare staff generates uncertainty and dissatisfaction with the care received.

*“He operated on me, he was late coming, and when he arrived, he said, ‘Oh, you are relieved because you are talking on your cell phone,’ and literally ‘I’m discharging you’ without examining me or anything, and I felt like, ‘What?’ He didn’t even ask me how I was doing, nothing, and he discharged me.” (CC1)*

For another patient, the opposite of compassionate care can be interpreted as the one-way communication established by some health professionals that limits their understanding and participation in the care and recovery process.

*“…Ah, yes, for example, the doctor who comes and knows that I have the fracture and doesn’t explain it to me well… he doesn’t specify well and just tells me that I have to operate and that’s it, and if you ask him a question, maybe they answer you or if not they leave you with the doubt and they don’t explain the situation of how you are” (CC 4, 11)*

The findings identified that one of the antonyms of compassionate care is reflected in the inadequate communication of some health professionals when delivering clinical diagnoses, characterized using derogatory terms and language lacking humanization, as interpreted from the testimony of one of the participants.

*“The doctor who diagnosed me with diabetes was very tough, the toughest in the whole world. We were in the emergency room, and he said to me: ‘Welcome to the world of diabetics’.” (JJ 9,10).*

## Discussion

This study explored the perceptions of 13 patients regarding the elements that constitute compassionate care, based on their relational experiences with healthcare staff during hospitalization. Four themes—healing presence, the experience of human connection, the holistic phenomenon of care, and the antonyms of care—were established as points of convergence between the patients' perceptions and the researchers' interpretations.

The meaning of “ Healing Presence,” as interpreted from the perceptions of four participants, aligns with the findings of the study conducted by Hermosilla-Ávila et al.^17^, which identified that the main strategies for meeting the comfort needs of palliative care patients were support, physical contact, affection, communication, knowledge, kindness, contact with nature, and contact with other people. The attitude adopted by healthcare staff can have a positive effect on the patient's well-being; the study concludes that non-pharmacological interventions, which may seem trivial and technologically simple, such as availability, affection, and support, have the capacity to significantly impact patients' comfort.

Similarly, another participant highlights close and compassionate communication as an element that fosters trust and tranquility in the recovery process. Cecconello et al.^18^ note that terminally ill patients experience physiological, psychosocial, and spiritual changes, requiring special care aimed at reducing suffering and improving quality of life, such as simple, frank, and honest communication and active listening. The authors indicate that healthcare personnel should offer comfort measures and care practices, but also be supported by an interdisciplinary team dedicated to promoting well-being. Thus, these results underscore the importance of implementing actions that improve communication within the healthcare team, which can help alleviate the physical and/or emotional suffering of patients.

Similarly, the Healing Presence was related to the levels of satisfaction that one of the patients had with the care received. In this regard, several studies have found that user satisfaction with the care provided by healthcare staff, specifically nursing staff, ranges between 60 and 90%, and define satisfaction as a state in which the brain produces a feeling of fullness, accompanied by rational security, producing in the patient a positive perception of the quality of care received^19^–^22^, Understanding that this transcends what happens in the therapist-patient relationship and implies changes and commitment in the organizational culture of health institutions and systems.

Compassion, for one of the participants, is an experience of human connection, which is reflected in the well-being and peace of mind that the healthcare staff provides to help patients cope with their in-hospital care. It has been found that, in care relationships focused on the well-being of the other, what is called“intelligent compassion” can emerge, a form of active empathy that combines sensitivity with clinical judgment^23^. Literature uses this concept to understand compassion and defines it as an attitude toward others that encompasses feelings, cognition, and behaviors centered on care, concern, tenderness, and an orientation toward supporting, helping, and understanding others, especially when suffering and need are perceived. Furthermore, a compassionate mindset includes attitudes and actions related to kindness, warmth, gentle treatment, and affection, among others^24^. Even from a neurobiological perspective, compassion is closely linked to limbic motivation and brain reward circuits, resulting in a profound and finely tuned self-regulation^6^; an experience considered highly effective for stress reduction, survival, and overall health^24^,^25^.

This study also revealed that for two of the participants, compassion is not limited to the satisfaction of basic needs but rather manifests as a holistic phenomenon of care that transcends institutional protocols and focuses on their overall well-being and that of their families. This result is consistent with findings from other studies, which have demonstrated that patient-centered care, considering their physical, emotional, and social needs, is associated with better clinical outcomes and greater satisfaction^26^, and that family involvement in patient care improves the quality of care and contributes to a faster and more successful recovery^27^.

The perceptions of care received by three of the participants were interpreted as antonyms of compassionate care, describing feelings of dehumanization, one-way communication, and misinformation from healthcare staff. Some studies^28^-^30^ have found a lack of closeness between healthcare staff and patients and/or caregivers, a distance that translates into a lack of emotional understanding and unmet needs, delays in care, and a lack of empathy. This phenomenon, also described as impersonal care, is a problem that negatively affects the patient experience and their perception of the quality of care received^31^. It leaves patients physically and psychologically vulnerable, generating a feeling of distrust toward the healthcare system and its professionals^29^. Furthermore, dehumanized care is associated with decreased patient satisfaction and reduced adherence to medical treatment, feelings of fear, insecurity and apprehension^30^,^31^.

While this is a novel study in the Spanish-speaking world, given its emphasis on patients' own perceptions of the care they received, it is important to consider its limitations. One limitation of the study is that it was conducted in a single healthcare institution where the staff is specifically trained in compassionate care and the humanization of care; therefore, patients may have a more positive perception of the care they receive. Furthermore, the heterogeneity and size of the sample, along with the fact that there was no opportunity to verify the transcribed information with the patients, can be considered a methodological limitation of the study.

## Conclusions

It was interpreted that compassionate care, or its absence, on the part of healthcare staff is clearly perceived by patients through the communicative relationship established. This finding opens a valuable space for research and management processes to be geared toward concrete improvements in the quality of care provided by the healthcare team.

Education and communication with patients are central elements of compassionate care, allowing for the establishment of meaningful and humanized relationships in care. The way healthcare staff convey information and interact with patients directly influences their perception of the care they receive.

The researchers explicitly acknowledge their reflexivity as researchers, recognizing that personal experiences and perspectives influenced the interpretation of the findings. Furthermore, they have articulated the emerging themes as interrelated phenomenological structures, which has allowed them to understand the participants’ lived experience in its complexity and depth, respecting its holistic meaning.
